# Identification and characterization of two CRISPR/Cas systems associated with the mosquito microbiome

**DOI:** 10.1099/acmi.0.000599.v4

**Published:** 2023-08-11

**Authors:** Shivanand Hegde, Hallie E. Rauch, Grant L. Hughes, Nikki Shariat

**Affiliations:** ^1^​ Department of Vector Biology and Tropical Disease Biology, Liverpool School of Tropical Medicine, Centre for Neglected Tropical Disease, Liverpool, UK; ^2^​ Department of Biology, Gettysburg College, Gettysburg, PA, USA; ^3^​ Department of Population Health, University of Georgia, Athens, GA, USA; ^‡^​ Present address: School of Life Sciences, University of Keele, Newcastle, UK

**Keywords:** *Anopheles*, CRISPR loci, microbiome, type I-F CRISPR, bacteriophage

## Abstract

The microbiome profoundly influences many traits in medically relevant vectors such as mosquitoes, and a greater functional understanding of host–microbe interactions may be exploited for novel microbial-based approaches to control mosquito-borne disease. Here, we characterized two novel clustered regularly interspaced short palindromic repeats (CRISPR)/Cas systems in *Serratia* sp. Ag1, which was isolated from the gut of an *Anopheles gambiae* mosquito. Two distinct CRISPR/Cas systems were identified in *Serratia* Ag1, CRISPR1 and CRISPR2. Based on *cas* gene composition, CRISPR1 is classified as a type I-E CRISPR/Cas system and has a single array, CRISPR1. CRISPR2 is a type I-F system with two arrays, CRISPR2.1 and CRISPR2.2. RT-PCR analyses show that all *cas* genes from both systems are expressed during logarithmic growth in culture media. The direct repeat sequences of CRISPRs 2.1 and 2.2 are identical and found in the arrays of other *Serratia* spp., including *S. marcescens* and *S. fonticola *, whereas CRISPR1 is not. We searched for potential spacer targets and revealed an interesting difference between the two systems: only 9 % of CRISPR1 (type I-E) targets are in phage sequences and 91 % are in plasmid sequences. Conversely, ~66 % of CRISPR2 (type I-F) targets are found within phage genomes. Our results highlight the presence of CRISPR loci in gut-associated bacteria of mosquitoes and indicate interplay between symbionts and invasive mobile genetic elements over evolutionary time.

## Data Summary

All the methods and data required for the reproduction of this work have been provided here. The accession numbers for the genomes of the bacterial isolates used in this study are JQEI00000000 (Serratia sp. Ag1) and JQEJ00000000 (Serratia sp. Ag2). No supporting external data were generated for this work.

## Introduction

Host-associated microbes play a crucial role in the physiology, diseases and immunity of their host. In mosquitoes, gut-associated microbes profoundly affect their host and these altered phenotypes influence vectoral capacity and vector competence [1–6]. Bacteria are abundant constituents of the gut microbiome of mosquitoes [7–10], but metagenomic studies have also found bacteriophage associated with these vectors [8–12], and it would be reasonable to expect interplay between these microbes, given their co-occurrence. While microbe–microbe interactions within the gut alter bacterial community structure and colonization [13, 14], less is known regarding the interactions between bacterial communities and bacteriophage, although signatures of these encounters can be inferred from bacterial genomes.

Clustered regularly interspaced short palindromic repeats (CRISPR)/Cas systems are present in approximately 45 % of sequenced bacterial genomes, and 90 % of archaeal genomes [15]. In their canonical function, they act as a small RNA-driven adaptive immune system that provides defence against exogenous nucleic acids, namely bacteriophage and plasmids [16, 17]. CRISPR/Cas systems have two components, a suite of *cas* genes and a CRISPR array [18]. The latter comprise direct repeat sequences ranging from 21 to 48 nucleotides in length that separate highly variable spacer sequences of similar lengths [19]. Spacers are commonly derived from foreign nucleic acids and are added in a polar manner to the CRISPR array, with the newest spacers being found closest to the leader sequence, which is directly upstream of the first repeat containing regulatory elements necessary for adaptation.

CRISPR immunity takes place in three distinct steps. First, new spacers are acquired and added to the array as the prokaryote adapts to a new invader [16, 20, 21]. Second, the array is transcribed, and the resulting transcript processed to produce mature CRISPR RNAs (crRNAs) [21, 22]. Third, the crRNA guides an endonuclease to its complementary target nucleic acid, thereby resulting in degradation, or interference, of the target [17, 21]. Various *cas* gene products are required for each of these steps.

CRISPR/Cas systems can be separated into two distinct classes and into further subtypes, depending on the complement and organization of *cas* genes [23]. Class 1, type I systems are defined by the inclusion of Cas3 as the effector endonuclease responsible for cleaving target DNAs. Within type I, there are seven subtypes, I-A–I-G [24–26]. Subtypes I-E (e.g. found in *Escherichia coli* and *Salmonella enterica *) and I-F (e.g. found in *Yersinia pseudotuberculosis* and *Pectobacterium atrosepticum *) differ slightly from each other. Type I-E has a distinct Cas2 protein, whereas in type I-F, Cas2 and Cas3 form a chimeric protein. Further, type I-F systems also lack Cas11, which forms part of the type I-E effector complex [22, 27]. In the family *Enterobacteriaceae *, CRISPR/Cas systems belong almost exclusively to either type I-E or type I-F [22, 27].

In addition to their well-characterized role in prokaryote adaptive immunity, alternative functions have also been attributed to some CRISPR/Cas systems [20]. These include roles in biofilm formation, host avoidance and symbiosis, and highlight the important biological roles of these systems in pathogenic bacteria, as well as other bacterial species [20, 28]. Given this, and the recent explosion in genome editing capabilities of *cas* genes, there is a drive to discover new CRISPR/Cas systems in a wide array of prokaryote genomes. CRISPR/Cas systems in host-associated microbiomes have mainly been examined in the context of human and plant microbiomes [29–32], while investigations in invertebrates are lacking. Studies focused on bacteria that play integral roles in the human microbiome have revealed important roles for CRISPR/Cas in viral resistance and mitigation of foreign genetic material [32–35]. Although CRISPR/Cas technology has been applied for genome editing of mosquito vector hosts and their microbiomes [36–38], characterizing native CRISPR loci in the gut bacteria of mosquitoes has not been attempted so far.

To determine interactions between the gut-associated bacteria of mosquitoes and bacteriophage over evolutionary time, we examined the genomic signature of CRISPR/Cas systems in Ag1, a *Serratia* strain previously isolated from *Anopheles gambiae* mosquitoes [39]. We found that Ag1 harbours two type I CRISPR systems and further classification revealed that they belong to subtypes I-E and I-F. We also examined the origins of the spacer region, thereby identifying past infections of the bacterial host, and characterized the expression of the *cas* genes. Our results indicate the presence of CRISPR/Cas systems in symbiotic bacteria associated within invertebrates and highlight the complexity of microbial interactions within the mosquito gut.

## Methods

### Culturing and nucleic acid isolation

The origins of the bacterial isolates *Serratia* sp. Ag1 and *Serratia* sp. Ag2 [JQEI00000000 (Serratia sp. Ag1) and JQEJ00000000 (Serratia sp. Ag2)] used in this study were described previously [39]. Total genomic DNA was isolated from overnight cultures of Ag1 and Ag2 using the Genome Wizard kit (Promega, WI, USA) and following the manufacturer’s protocol. DNA pellets were resuspended in 200 µl of molecular grade water and stored at −20 °C. Bacterial strains were cultured in LB broth to log phase and to stationary phase and total RNA was isolated using TRIzol (Life Technologies, CA, USA) and resuspended in 20 µl molecular-grade water. RNA was treated with 1-unit DNase (Life Technologies, CA, USA) and reisolated with TRIzol. Pellets were resuspended in 20 µl molecular grade water and stored at −20 °C.

### RT-PCR expression analyses

A total of 100 ng total RNA was used to generate cDNA in a 20 µl reaction using a qScript mastermix (QuantaBio, MA, USA) that contained random hexamers. Reverse transcription was performed in a PCR machine with the following parameters: 22 °C for 5 min, 42 °C for 30 min, 85 °C for 5 min and 4 °C hold. For a non-RT control, reactions were set up in duplicate but without RT enzyme. The cDNAs were diluted 1 : 10 and 2 µl of each was used for subsequent PCR reactions with one unit of *Taq* polymerase (New England Biolabs, MA USA), 200 uM dNTPs (New England Biolabs, MA, USA) and 1× standard *Taq* polymerase buffer in a 25 µl reaction. The primers used for RT-PCR analysis of *cas* genes are listed in Table 1. Following initial denaturation for 3 min at 95 °C, the PCR conditions were as follows: 20 cycles (16S control PCR) or 25 cycles (*cas* genes) of 95 °C for 30 s, annealing at 57 °C for 30 s and an extension at 72 °C for 30 s. A total of 5 µl of the PCR reaction was imaged by gel electrophoresis. The RT-PCR experiments were run independently twice (different bacterial cultures), and on one of these, the PCR step was performed twice on the same cDNA. The gel (Fig. 4) is from one of the independent experiments. Densitometry was not performed as the difference between the log and stationary phases was clear.

**Table 1.** Primers used in this study

	Marker	Orientation	Primer sequence (5′–3′)	Annealing temp. (°C)
Type I-E	*cas3*	Forward	GCTAATCTCACGATGCAACTGC	58
		Reverse	CATATAAGGCCGCCTCGGT	58
	*cse1*	Forward	TGGTAATGTATCCAACGCTGGG	58
		Reverse	ATGCCGTTATCCGCCAACAG	58
	*cse2*	Forward	CAAGTTCTCTAGAGCCGAACGA	58
		Reverse	CCATTGTGGGGTTGTCTGCT	58
	*cas6e*	Forward	AATTTCAAGACAAGATTGGCCAACA	58
		Reverse	GCCCTTGCCAATACCATGTTTAAAG	58
	*cas7*	Forward	GCCGCCATGTTAACCAATGAG	58
		Reverse	CCATCGCCTCACCACATTGAG	58
	*cas5*	Forward	ATGGCTGGCGCAAATGAATG	58
		Reverse	CCACCATCTGAAAGTCACGCA	58
	*cas1*	Forward	GGAATGGAAGGTAATCGTGTTCGT	58
		Reverse	TTGGTCAGATCACTCAGCTGAAAT	58
	*cas2*	Forward	AAATGACTTACCACCTGCTGTTC	58
		Reverse	CTCTGTCGGAGAATATTGCATCAAG	58
	CRISPR1_sp1	Forward	TTTCTGCCTCCGCGCCAT	60
	CRISPR1_sp3	Forward	TTCTGTGGTCGTCGTCAGTACO	60
	CRISPR1_sp7	Forward	TTCTCTTAGGGTGCCTGCGC	60
	CRISPR1_sp1_rev	Forward	AAGACTCTGCCGGTAGCGG	60
	CRISPR1_sp3_rev	Forward	GGAAGACGTTTCAGAATATGCGGTA	60
Type I-F	*cas1*	Forward	ATTGCCGCATTCTGGTTAACG	58
		Reverse	CAGCATCACTGCCGTGGTATT	58
	*cas3*	Forward	GCTCTACAACGGTGCAGGAT	58
		Reverse	TCTTGCCACTTTTCCGTCGC	58
	*csy1*	Forward	CAGATCAGCCTGGTGACTCAC	58
		Reverse	TTCAACGCCAATGTGGAGAGATAG	58
	*csy2*	Forward	ATTTCTGGCGGTGAAGCAGG	58
		Reverse	CCTGTAGCCCGTTAATCGTCC	58
	*csy3*	Forward	CGACGCCGTCTACCTGTAAT	58
		Reverse	GCAATATTGGTGGCATAACGCC	58
	*cas6f*	Forward	CGTTTGAACAAATACCGGATACCCA	58
		Reverse	AATTCACCATGCTGAATATAAATTCGCATO	58
	CRISPR2_sp1	Forward	AAAGCAGCTGAAGCGTTGAAGC	60
	CRISPR2_sp4	Forward	ATGCGTCGGGTGAGCAACC	60
	CRISPR2_sp8	Forward	AAGCCATGGAACGTGCGGG	60
	CRISPR2_sp1_rev	Forward	AACGCTGGCCATCAGCTTCA	60
	CRISPR2_sp4_rev	Forward	ACAAACGCAGCAAAGAGGTTGC	60

### Identification of CRISPR loci, phylogenetic analyses and spacer identification

The assembled Ag1 genome was analysed using CRISPR-Finder [40] to identify both the CRISPR arrays and the *cas* genes. We used the default setting to analyse the Ag1 genome to identify the CRISPR array and *cas* genes. Spacers were extracted from the arrays and analysed using an Excel-based macro [41]. CRISPR Target [42] was used to identify putative spacer matches. Here, we used default parameters for the initial blast screen and target database. For initial output display parameters, we used a default score cut-off of 20, 26/32 base pairs. We considered matches to be 24/32 or 24/33 nucleotides for the type I-E and I-F spacers, respectively. For phylogenetic analyses, the coding sequences of both *cas3* genes were translated and blast was used to find the top 20 similar sequences from different species. These amino acid sequences were used in mega 7 to build phylogenetic trees with a bootstrap value of 1000 [43].

## Results

**Fig. 1.**
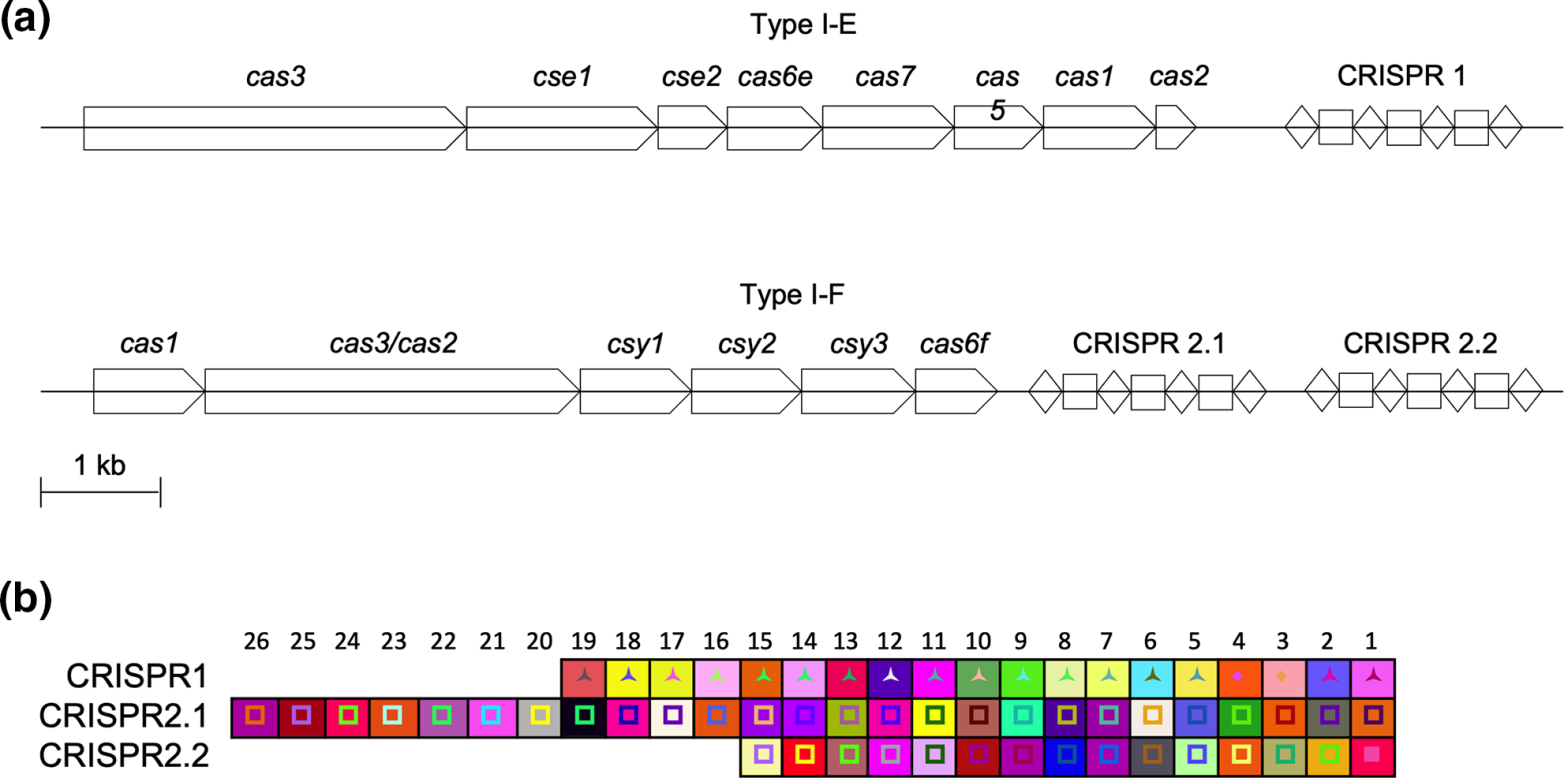
Organization and expression of the type I-E and type I-F CRISPR/Cas systems of *Serratia* sp. Ag1. (a) All *cas* genes are shown in the forward orientation. Direct repeats in the CRISPR array are shown as black diamonds, while the spacer sequences are represented by white squares. The *cas* genes are scaled to the 1 kb bar shown in the bottom left. (b) Spacer composition of the three CRISPR arrays in Ag1. The unique combination of the background colour and the shape and colour in the foreground represents a single spacer sequence. The three-point star represents a spacer that is 33 nt in length. The inner square represents a 32 nt spacer. The oldest spacer (spacer number 1) is shown to the far right, while the most recently acquired spacer is shown on the far left. The invariant direct repeats have been removed for clarity.

We identified two type I CRISPR/Cas systems in Ag1 and termed them CRISPR1 and CRISPR2. The former has a single CRISPR array and is of the type I-E subtype of CRISPR/Cas systems (Fig. 1a), with direct repeats and spacers that are 28 and 33 nucleotides long, respectively. The CRISPR2 has a *cas* operon associated with the type I-F subtype, and there were two CRISPR arrays associated with this system, which we termed CRISPR2.1 and CRISPR2.2. The direct repeats and spacers in both arrays are 28 and 32 nucleotides in length, respectively. The type I-E repeat sequences fall under cluster 2 and the type I-F direct repeat sequences fall under cluster 1 [44]. These cluster designations follow those described in [44]. The spacer composition of the three CRISPR arrays in Ag1 was analysed and the spacer content of each array was distinct (Fig. 1b). CRISPR2.1 was the longest array and contained 26 different spacers.

**Fig. 2.**
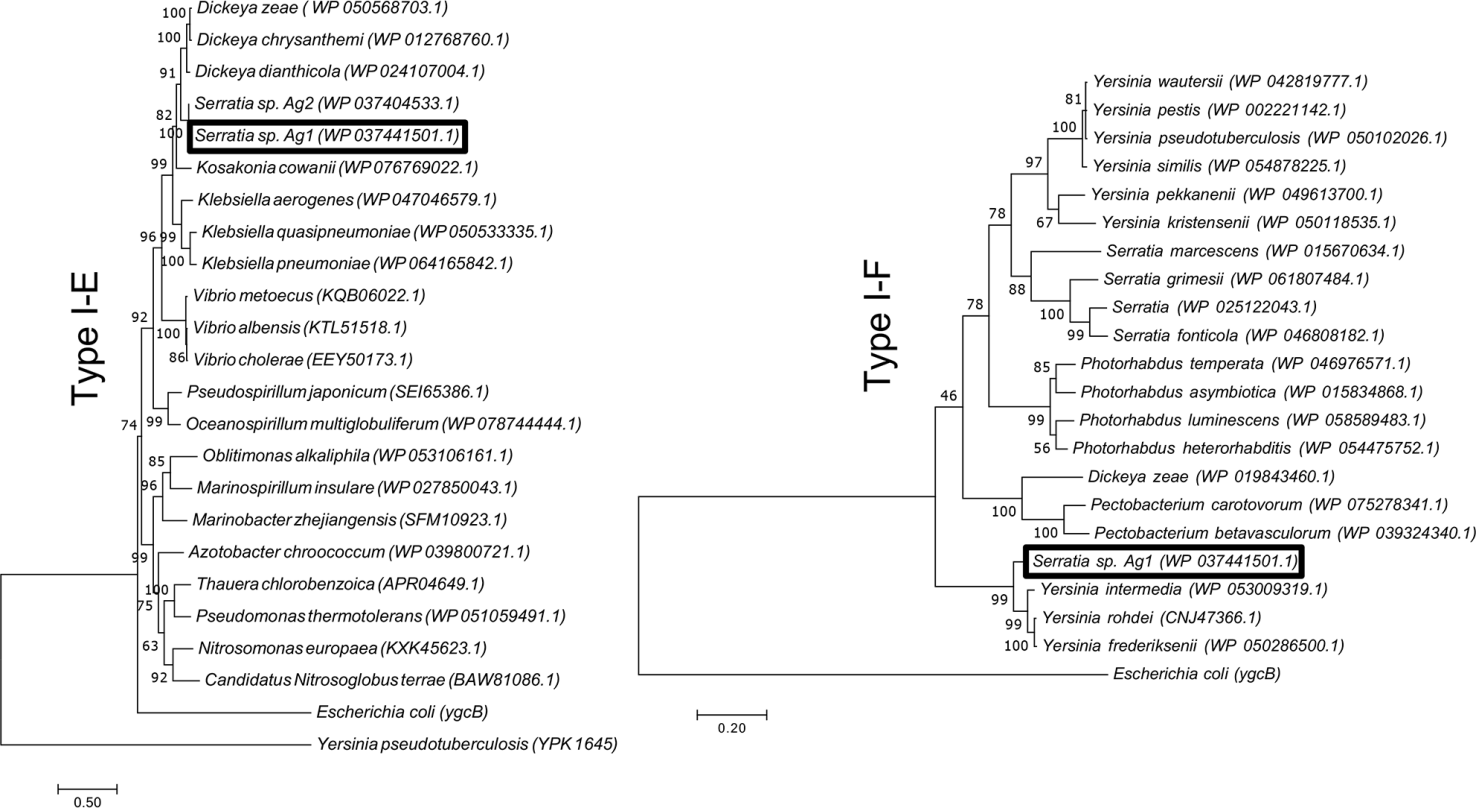
Phylogenetic analyses of type I-E and type I-F Cas3 from *Serratia* sp. Ag1. Phylogenetic trees show analyses of Cas3 with the top 20 closest blastp hits for both trees. Maximum-likelihood trees based on the relevant Cas3 protein are shown with a bootstrap value of 1000. *E. coli* is included as a representative of the type I-E subtype, and *Y. pseudotuberculosis* is included as a representative of type I-F.

Using the cas3 protein sequence from each CRISPR/Cas system, we identified similar protein sequences from other bacterial species and examined their phylogeny. We found a single match to another *Serratia* sp. Ag2, which is closely related to Ag1 [39] (Fig. 2). Otherwise, we did not find any other *Serratia* spp. whose cas3 matched closely to the cas3 of CRISPR1, suggesting that the type I-E system is not broadly present in other *Serratia* spp. The type I-E cas3 was closely related to *Dickeya* spp. and *Klebsiella* spp., and overall there was little divergence among the type I-E cas3 proteins compared to those from the type I-F subtype (Fig. 2). Conversely, we found several *Serratia* spp. that contained cas3 protein sequences of the type I-F subtype, although the sequence from Ag1 was more closely related to some *Yersina* spp. than those *Serratia* spp.

**Fig. 3.**
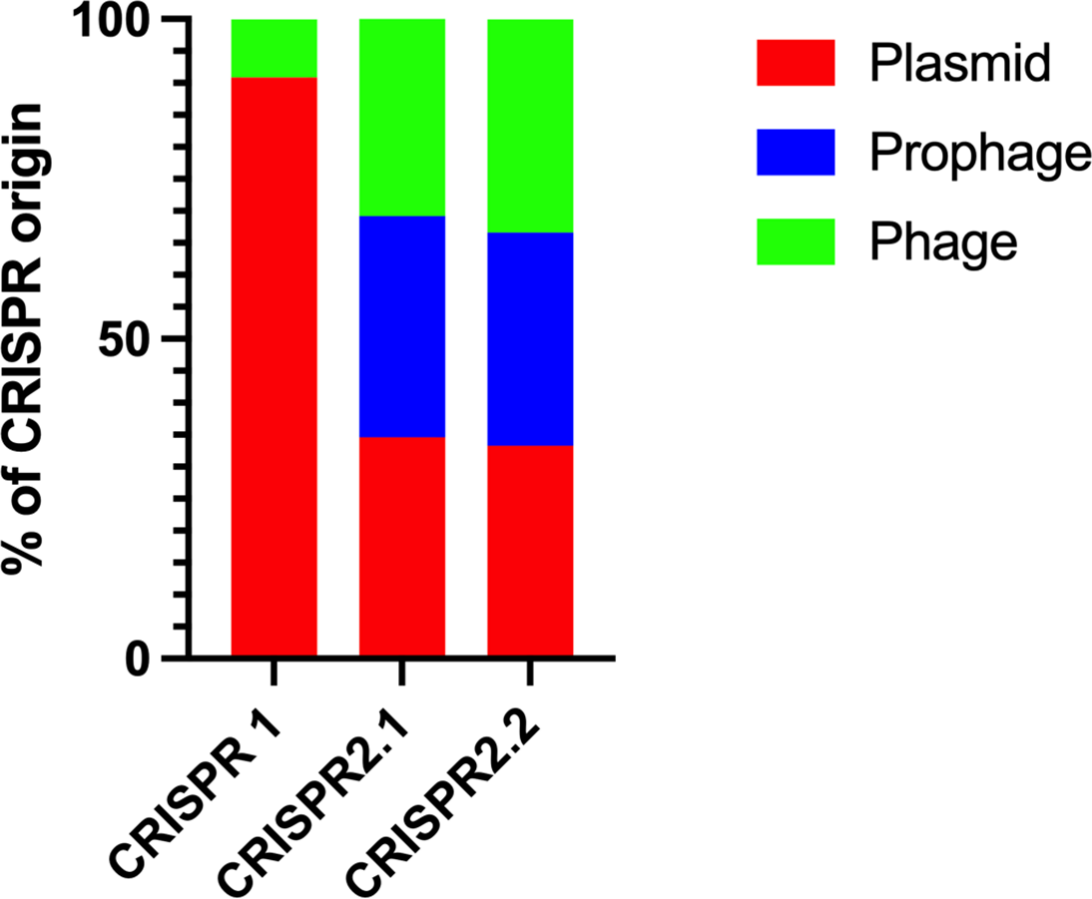
Origin of exogenous nucleic acid elements in the CRISPR loci: percentage of plasmids, phage and prophage DNA found in the spacer sequences for each CRISPR array in *Serratia* sp. Ag1.

**Fig. 4.**
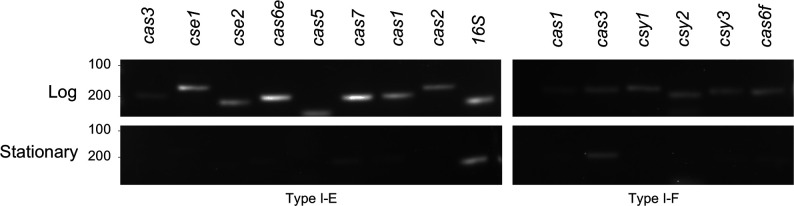
Expression of the type I-E and type I-F *cas* genes. The RT-PCR analysis of expression of *cas* and *csy* genes in logarithmic and stationary growth phase of *Serratia* sp. Ag1. A 100 base pair ladder was used, and sizes are indicated to the left of the gel images.

We analysed the CRISPR spacers to determine whether they matched to any exogenous nucleic acids and found a greater number of matches to plasmid and bacteriophage (including prophage sequences) sequences in CRISPR2.1 (50 %, 13/26 spacers had matches) and CRISPR2.2 (53 %, 8/15) than in CRISPR1 (32 %, 6/19) (Fig. 3, Table 2). In both CRISPR2.1 and 2.2, phage targets accounted for the most hits, constituting two-thirds of the identified targets (Fig. 3). Conversely, CRISPR1 had fewer spacer targets that we could identify, with most identified targets of plasmid origin (Fig. 3). When we increased the stringency of the matches to 85 % (28/33 nucleotides for the CRISPR1 array, 27/32 for CRISPR2 arrays), the number of hits decreased significantly. Of the remaining 23 spacer targets, only 1 matched to a spacer in CRISPR1 and 22 spacers matched to a phage target. Expression of the *cas* genes from both subtypes was analysed by RT-PCR and for both subtypes the expression of all *cas* genes was greater during log growth than in stationary phase (Fig. 4).

**Table 2.** Protospacer identity: origin of protospacer sequences matched against plasmids, phage and prophage

	Spacer	Target species	Organism	Protein target	Protein ID	Nucleotide ID	Nucleotide ID (percentage)
CRISPR1	19	Plasmid	* Rhizobium leguminosarum* bv*. viciae* plasmid pRL8	Aminotransferase class V-fold PLP-dependent	WP_011654699.1	25/33	75.76
		Plasmid	* Azospirillum brasilense* strain Az39 plasmid	Helix–turn–helix domain-containing protein	WP_040134386.1	26/33	78.79
		Plasmid	* Arthrobacter* sp*.* ERGS1 : 01 plasmid unnamed2	DUF3416 domain-containing protein	WP_082368864.1	24/33	72.73
		Plasmid	* Neorhizobium galegae* chromid pHAMBI540a	DUF1211 domain-containing protein	WP_035996019.1	25/33	75.76
	16	Phage	AJ564013_AJ564013 bacteriophage PY54	Immunity repressor Icd	CAD91791.1	28/33	84.85
	10	Plasmid	* Escherichia coli* strain 08–00022 plasmid pCFSAN004179G	Conjugative transfer relaxase/helicase trai	WP_047088735.1	27/33	81.82
	7	Plasmid	* Enterococcus faecium* strain E1 plasmid	Glycoside hydrolase family 1 protein	WP_002289584.1	26/33	78.79
		Plasmid	* Burkholderia caribensis* MBA4 plasmid	DUF1211 domain-containing protein	WP_035996019.1	25/33	75.76
		Plasmid	* Enterococcus faecium* DO plasmid 3	Beta-glucosidase	YP_006377528.1	26/33	78.79
	5	Plasmid	* Cupriavidus metallidurans* CH34 megaplasmid	Anion permease	WP_011518338.1	26/33	78.79
	1	Plasmid	* Sinorhizobium fredii* NGR234 plasmid	Hypothetical protein	NP_443999.1	24/33	72.73
CRISPR2.1	26	Phage*	* Klebsiella pneumoniae* strain Kp_Goe_149832 plasmid	Phage tail tape measure protein	WP_048292313.1	24/32	75.00
		Plasmid	* Shigella boydii* CDC 3083–94 plasmid	Type II toxin–antitoxin system	WP_000604847.1	25/32	78.13
		Plasmid	* Methylobacterium radiotolerans* JCM 2831 plasmid	MFS transporter	WP_012329600.1	24/32	75.00
		Phage	AF226852_AF226852 *Pseudomonas* phage phi8	P10	AAF63303.1	24/32	75.00
	26	Prophage	* Serratia plymuthica* 4r×13	Phage tail tape measure protein	WP_041417117.1	28/32	87.50
	25	Phage*	* Anoxybacillus amylolyticus* strain DSM 15939 plasmid	DNA primase	WP_084256491.1	25/32	78.13
	24	Prophage	* Yersinia mollaretii* strain IP25089	Baseplate protein	WP_049611672.1	30/32	93.75
	23	Prophage	* Serratia marcescens* strain 907_SMAR 425_37092_653157	Recombinase	WP_049202623.1	29/32	90.63
	21	Phage	KT898134_KT898134 *Aeromonas* phage phiARM81mr	Terminase large subunit	ALN97629.1	31/32	96.88
		Plasmid	* Synechococcus* sp*.* PCC 7117 plasmid unnamed5	Type I-D CRISPR-associated endonuclease Cas1	WP_065712013.1	26/32	81.25
		Prophage	* Aeromonas hydrophila* strain Ah-HSP	Terminase	WP_077096195.1	31/32	96.88
		Plasmid	* Rhodobacter sphaeroides* ATCC 17025 plasmid	Gluconate : proton symporter	WP 011910413.1	25/32	78.13
	18	Phage	EU307292_EU307292 *Burkholderia* phage Bups phi1	Putative PAPS reductase/sulfotransferase	ABY40518.1	27/32	84.38
		Plasmid	* Klebsiella oxytoca* strain 2880STDY5682598	Hypothetical protein	WP_064405428.1	29/32	90.63
	17	Plasmid	* Shigella flexneri* 1 a strain 0228 plasmid	Hypothetical protein	NZ_CP012733.1	26/32	81.25
		Prophage	* Cronobacter sakazakii* strain 699	Phage protein	WP_007882954.1	30/32	93.75
	16	Phage*	* Streptomyces* sp. F2 plasmid	Hypothetical protein	YP_008996313.1	26/32	81.25
	12	Phage	KY709687_KY709687 *Salmonella* phage 29 485	Hypothetical protein	arb10913.1	27/32	84.38
	9	Plasmid	* Yersinia pseudotuberculosis* IP 32953 plasmid	Type IA DNA topoisomerase	WP_011191426.1	26/32	81.25
	7	Prophage	* Serratia marcescens* 2880STDY5682985	Hypothetical protein	WP_060433331.1	29/32	90.63
		Prophage	* Klebsiella oxytoca* strain 2880STDY5682691	Transcriptional regulator	NZ_FKZE01000009	29/32	90.63
		Prophage	* Sodalis glossinidius* str. morsitans	Hypothetical protein	WP_041867073.1	29/32	90.63
		Plasmid	* Sphingomonas sanxanigenens* DSM 19645	Hypothetical protein	WP_075153410.1	24/32	75.00
	5	Prophage	* Serratia* sp. S4	DUF968 domain-containing protein	WP_017892522.1	31/32	96.88
		Phage	JQ182729 JQ182729 *Enterobacterial* phage mEp390	Hypothetical protein	AFM76141.1	27/32	84.38
		Plasmid	* Dinoroseobacter shibae* DFL 12 plasmid	FAD-dependent oxidoreductase	WP_012187434.1	24/32	75.00
CRISPR2.2	15	Plasmid	* Alteromonas mediterranea* strain CP48 plasmid	Single-stranded DNA-binding protein	WP_071960856.1	27/32	84.38
		Plasmid	* Kangiella geojedonensis* strain YCS-5	Single-stranded DNA-binding protein	WP_046560643.1	29/32	90.63
	13	Phage*	* Methylomonas* sp. DH-1 plasmid	Atp/GTP-binding protein	NP_639742.1	26/32	81.25
		Prophage	* Yersinia aldovae* strain IP23238	Phage capsid protein	WP_049687658.1	29/32	90.63
	8	Phage*	* Klebsiella pneumoniae* strain Kp_Goe_827024 plasmid	Histidine kinase	WP_064023699.1	26/32	81.25
		Prophage	* Klebsiella pneumoniae* strain CHS159	Tail assembly protein	WP_016530340.1	30/32	93.75
	7	Phage*	* Pantoea* sp. At-9b plasmid	Hypothetical protein	WP_013511365.1	29/32	90.63
		Prophage	* Pantoea* sp. At-9b plasmid pPAT9B01	Hypothetical protein	WP_013511365.1	29/32	90.63
	6	Phage*	* Streptomyces coelicolor* A3(2) plasmid	Atp/GTP-binding protein	NP_639742.1	26/32	81.25
	5	Prophage	* Serratia marcescens* strain 2880STDY5682949	Phage head protein	WP_060427589.1	29/32	90.63
	4	Plasmid	*Microscilla sp. pre1 plasmid*	Ms149	NP_116837.1	26/32	81.25
	1	Plasmid	* Rahnella* sp. J11-6	Noncoding		31/32	96.88

*Denotes that the organism is listed as a plasmid in the NCBI database but is suspected to be a phage due to protein content.

## Discussion

Bacteria living in complex ecological settings are continuously challenged by predatory viruses. The CRISPR/Cas adaptative immune systems of bacteria protect bacteria from some of these challenges by targeting foreign genetic material such as plasmids and bacteriophage [16, 45]. Here, we provide evidence of CRISPR/Cas systems in the mosquito-associated *Serratia* sp. Ag1, which was isolated from *Anopheles gambiae* [39]. We have identified two type I CRISPR/Cas systems, which are typically found in the family *Enterobacteriaceae* [22, 27]. CRISPR/Cas systems in *Serratia marcescens* have been described previously, and most strains harbour a type I-F or both a type I-E and a type I-F system [27, 34, 46, 47]. One *Serratia* sp. (ATCC39006) contains both these type I systems and also a type III-A system [48].

Analysis of CRISPR spacer sequences in Ag1 confirmed the origin of many spacer sequences. Our results revealed hat 47 % (23/49) of the spacer targets that we could identify originated from plasmids, while bacteriophage (phage and prophage) accounted for two-thirds of the matched spacers. Overall, 53 % (26/49) of spacers matched to phage or plasmid sequences. This is higher than for other *Enterobacteriaceae *, such as *Salmonella* (12%), *E. coli* (19%) and Shiga toxin-producing *E. coli* (8 %) [49] . Extensive spacer sequence analysis has been performed in the genomes of *Enterobacteriaceae *, which are human pathogens and commensals [27, 50–52]. A discrete number of spacers from the *Enterobacteriaceae* members appear to be acquired from extrachromosomal genetic elements, such as plasmids and bacteriophage, while other spacers match to the bacterial host genome in non-prophage regions, although many of the spacers are still of unknown origin [27].

Cas proteins play crucial roles in all three steps of CRISPR/Cas immunity [21, 53]. Our results showed active expression of all *cas* genes during the actively dividing logarithmic growth phage of bacteria and attenuation of all but the type I-F *cas3* gene during stationary phase. This is concordant with previous studies in *E. coli* showing repression of the type I-E *cas3* gene expression during stationary phase compared to log phase [54–56]. Our results for expression analyses of type I-F *cas* gene show continuous expression even in the stationary phase. This result is similar to what was reported in the phytopathogen *Pectobacterium atrosepticum* showing expression of Cas protein in both exponential and stationary growth stages [57, 58]

CRISPR spacer sequences can be used for bacterial subtyping [59]. The presence of the type I-F cas3 in multiple *Serratia* spp. suggests that these genomes likely also contain CRISPR arrays. This would depend on CRISPR arrays being present in all strains of the species and exhibiting strain-to-strain variability that could be exploited for subtyping. Whole-genome sequencing of four *Serratia marcescens* genomes showed that CRISPR/Cas systems were absent in half of these [34]. The prevalence of CRISPR/Cas systems and the diversity of spacer content in other *Serratia* spp. is yet to be determined and would need to be performed to determine the utility of CRISPR typing in this bacterium.

While Cas1 and Cas2 are mainly involved in acquiring the spacers from newly invading phage and foreign genetic material, the cas2/3 ; csy complexes are involved in the priming method for spacer acquisition [60, 61]. Our results show the presence of newly acquired spacer sequences, suggesting that adaptation is occurring actively in these bacteria. Hence, there is the possibility of recurrent encounters between phage and symbiotic bacteria in the mosquito gut. A recent study demonstrated that phage infection can alter bacterial levels in mosquitoes and alter their development in aquatic stages [62]. These phage may also be part of the mosquito gut microbiome, where they interact with gut bacteria and compete for nutritional resources.

The CRISPR/Cas system in bacteria has been explored extensively in terms of its application in different fields, such as human and agriculture diseases [16, 21, 63, 64]. However, analysis of CRISPR loci in the host-associated symbiotic bacteria is limited, especially the role of CRISPR systems in the host–microbe interactions. Apart from anti-viral defence, CRISPR has been shown to be involved in DNA repair, colonization and host immune evasion [65–67]. Hence, by modifying the CRISPR loci the colonization of bacteria in the host environment could be investigated. Such studies are important in deciphering the host–microbe interactions in complex ecological settings such as the mosquito microbiome. In this regard, further studies are needed to analyse CRISPR loci in the mosquito symbionts and understand the mechanistic basis for CRISPR loci-mediated host–microbe interactions.
